# Physician Trainees’ Decision Making and Information Processing: Choice Size and Medicare Part D

**DOI:** 10.1371/journal.pone.0077096

**Published:** 2013-10-07

**Authors:** Andrew J. Barnes, Yaniv Hanoch, Melissa Martynenko, Stacey Wood, Thomas Rice, Alex D. Federman

**Affiliations:** 1 Healthcare Policy and Research, Virginia Commonwealth University, Richmond, Virginia, United States of America; 2 School of Psychology, University of Plymouth, Plymouth, United Kingdom; 3 General Internal Medicine, Mount Sinai School of Medicine, New York, New York, United States of America; 4 Psychology, Scripps College, Claremont, California, United States of America; 5 Health Policy and Management, University of California Los Angeles, Los Angeles, California, United States of America; University of St Andrews, United Kingdom

## Abstract

Many patients expect their doctor to help them choose a Medicare prescription drug plan. Whether the size of the choice set affects clinicians’ decision processes and strategy selection, and the quality of their choice, as it does their older patients, is an important question with serious financial consequences. Seventy medical students and internal medicine residents completed a within-subject design using Mouselab, a computer program that allows the information-acquisition process to be examined. We examined highly numerate physician trainees’ decision processes, strategy, and their ability to pick the cheapest drug plan—as price was deemed the most important factor in Medicare beneficiaries’ plan choice—from either 3 or 9 drug plans. Before adjustment, participants were significantly more likely to identify the lowest cost plan when facing three versus nine choices (67.3% vs. 32.8%, p<0.01) and paid significantly less in excess premiums ($60.00 vs. $128.51, p<0.01). Compared to the three-plan condition, in the nine-plan condition participants spent significantly less time acquiring information on each attribute (p<0.05) and were more likely to employ decision strategies focusing on comparing alternate plans across a single attribute (search pattern, p<0.05). After adjusting for decision process and strategy, numeracy, and amount of medical training, the odds were 10.75 times higher that trainees would choose the lowest cost Medicare Part D drug plan when facing 3 versus 9 drug plans (p<0.05). Although employing more efficient search strategies in the complex choice environment, physician trainees experienced similar difficulty in choosing the lowest cost prescription drug plans as older patients do. Our results add further evidence that simplifications to the Medicare Part D decision environment are needed and suggest physicians’ role in their patients’ Part D choices may be most productive when assisting seniors with forecasting their expected medication needs and then referring them to the Medicare website or helpline.

## Introduction

The Medicare Prescription Drug, Improvement, and Modernization Act of 2006 (Medicare Part D) is one of the most important changes to the U.S. health care system. Under the program, millions of Medicare beneficiaries were afforded the opportunity to purchase subsidized prescription drug coverage. However, Medicare Part D is not without its critics. One of the major criticisms—voiced by beneficiaries, pharmacists, and physicians [1]—focuses on the choice-rich design of the program. Indeed, in 2009 there were over 45 plans to choose from in every state [2]. As of 2013, most states offer 30 or more plans [3]. The program is further complicated by the fact that drug plans differ along a range of important features, such as drug formularies and cost-sharing requirements. Therefore, decisions current beneficiaries face about which plan to purchase or switch into involves comparing over 30 plans along six attributes. (As of April 2, 2013, the attributes listed on the official Medicare website were plan name, estimated annual cost, monthly drug premium, annual deductible, drug coverage/drug restrictions, and overall plan rating. At the time of this study, “coverage in the gap” and “number of network pharmacies” were used in place of “drug coverage/drug restrictions” and “overall plan rating.”) Indeed, evidence suggests that beneficiaries’ have difficulty choosing the least expensive drug plans for their medication needs oftentimes resulting in financial and health consequences [4–6]. A series of recent studies from the field and the laboratory indicate seniors are facing choice overload in the Medicare Part D market and may fare better if confronted with fewer drug plan options and more assistance in their decision making [6–8].

From Medicare Part D’s early days, it was assumed that one way beneficiaries could overcome the complexity of the program would be by consulting their clinicians, who are most knowledgeable about their health care needs and prescription usage. In fact, the American Academy of Family Physicians [9], the popular press [10], as well as seniors themselves [11,12] expected doctors should be well-versed about the program and help their patients make a decision. These expectations resonate well with a study [13] showing that older adults trust physicians (and pharmacists) more than any other source to supply them with information about prescription drug prices and effectiveness.

After 6 years, have older adults become familiar with the program, and do they seek help from other sources? No recent data can speak directly to these questions, but earlier work [14] found that many older beneficiaries had restricted knowledge about vital features of the Part D program. For example, fewer than half (40%) knew about the nature of the cost sharing of their drug plan or that their drug plan offered coverage in the gap. Furthermore, even though Medicare offers a range of resources (e.g., website, toll-free help lines) to aid older adults in choosing a drug plan, relatively few people use them [15]. These results are not isolated. Earlier investigations examining older adults’ knowledge about hospital and physician components of the Medicare program found that even though these coverage programs had been around for many years, older adults exhibited only limited knowledge of it [16], and one survey [17] revealed that over 25% of beneficiaries were not even aware of Medicare’s annual open enrollment period. Finally, although older adults have indicated that saving money is the most important factor in their decision about which drug plan to purchase [15], only a small minority actually choose the lowest cost plan available to them [4]. In fact, in the most up-to-date analysis of beneficiaries’ claims, researchers [6] found that only 5.2% had picked the cheapest plan.

Older Medicare recipients are not the only ones having these difficulties, however. Health care professionals have also had trouble navigating the new Medicare Part D, finding it difficult, for example, to identify which prescription drugs are covered by their patients’ plan formularies [18]. Given older adults’ difficulties in choosing a Part D plan and their expectations for assistance from health care providers, clinicians’ ability to navigate the Medicare Part D program successfully has strong economic and health implications. Whether clinicians’ decisions are affected by the number of drug plans is an important question.

How should we evaluate clinicians’ decision abilities in regard to the Medicare Part D program? Physician trainees represent an important population in which to evaluate decision making because they possess the essential cognitive skills to make an informed decision if given sufficient information about the drug plans, even though they have likely had little exposure to the drug plan choice in the field. Knowing whether physician trainees can adequately make these decisions can inform whether decision skills that would help them advise patients on choosing insurance plans given their health and financial status should be included in medical education curricula. An earlier study [19] examining physician trainees’ ability to choose the cheapest drug plan from 3, 10, or 20 different plans found that as choice size increased, their ability to pick the cheapest drug plan diminished. Although this study was informative, it was limited in its scope, failing to control for trainees’ decision-making processes and strategy selection. That is, it focused only on the decision outcome and not on the decision processes that could inform medical training.

One useful way to examine individuals’ decision-making processes, strategy selection, and decision quality is to employ a process-tracing method, such as Mouselab. Aside from allowing researchers to more closely imitate the real decision environment faced by future clinicians, Mouselab offers the ability to examine the information being sought, the time spent on each piece of information, and how the decision environment (e.g., three vs. nine plans) affects decision making. Thus, using Mouselab confers a key comparative advantage to earlier investigations. This is an important omission, as the strategies employed could influence the decision outcome for physician trainees as they do for older adults [7]. For example, we know that task complexity [20] and time pressure [21,22]—because of their influence on the cognitive system—could cause changes in decision strategies and quality. Increasing the number of drug plans from three to nine, therefore, could determine not only the decision outcome but, importantly, the decision-making process, as well.

Aside from examining a timely, policy-relevant issue, we augment previous research by (a) varying the number of Medicare drug plans physician trainees’ evaluated (either three or nine), (b) including objective outcome criteria—namely, whether trainees chose the cheapest drug plan (and the amount of money lost if a higher cost plan was chosen), and (c) controlling for decision process and strategy variables that may be important intermediaries of the choice-size effect found among clinical decision makers. We hypothesized that medical students and residents would employ less efficient search strategies as the choice became more complex (i.e., nine vs. three drug plans) and that a larger drug plan menu would lead to poorer performance—that is, not choosing the lowest cost plan—independent of amount of medical training (i.e., student or resident) and decision-making strategy.

## Methods

### Participants

Participants were medical students and internal medicine residents recruited at a major U.S. medical school. Our study was conducted online. All participants provided informed consent prior to participating in the study by reading the consent page online and clicking on a consent button before the survey would begin. The study was approved by Mount Sinai School of Medicine’s institutional review board and was conducted according to the principles expressed in the Declaration of Helsinki. Seventy-eight participants were enrolled in the study and 70 completed both the questionnaire and the decision trials. Of those completing the study, the average age was 25.7 years (SD 2.3), with more medical students than residents participating (86.1% vs. 13.9%).

### Measures and Procedure

#### Mouselab

Mouselab is a computerized information-tracing tool [20,22,23] that allows researchers to gain insight into the processes by which individuals make decisions by evaluating what information is being sought, the time spent processing information, and how different choice sets (e.g., three vs. nine drug plans) affect the decision strategy. Following a practice trial, participants read a hypothetical scenario about a friend, “Bill,” that was presented and all participants were asked to help Bill choose a Medicare prescription drug plan. More specifically, participants read the following paragraphs, and were asked to choose a Medicare drug plan based only on the information presented to them:

Imagine that one of your friends, whom we’ll call Bill, has asked you to help him in choosing a Medicare prescription drug plan. He has made it clear that he is not sure how to choose among the different drug plans, and therefore would like you to make the choice for him. However, Bill has told you a little about the type of drug plan he would like. He does not want to spend a lot of money. That is, he wants to keep his annual cost, monthly premium, and annual deductible as low as possible. He is, however, not sure whether he should get a plan that offers coverage in the gap. He is also interested in a company that he knows and feels he can trust. Finally, he expects to get all of his drugs by calling a toll-free phone number, and having them mailed to his home. In the screens that follow, you will see information about a range of drug plans (their name, their estimated annual cost, their monthly drug premium, the number of network pharmacies, whether they offer coverage in the gap, and their annual deductible). Please try to make the best choice for Bill.

**Figure 1:**
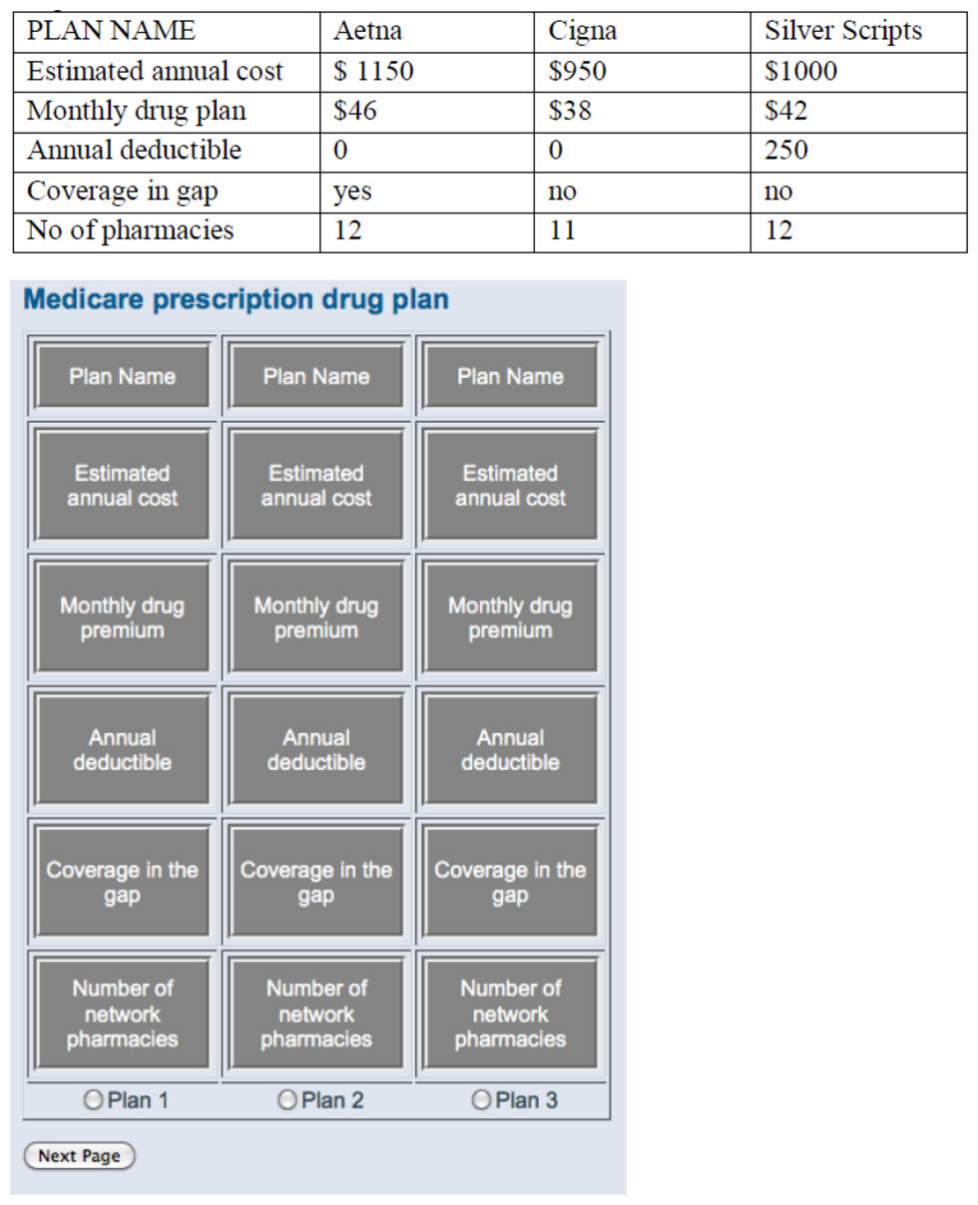
Medicare Part D Decision Task and Mouselab Screenshot. A. Medicare Part D Decision Task.

After reading a scenario description on the Medicare prescription drug plan, participants were instructed to choose the prescription drug plan that suits Bill’s needs. Information about drug plans—taken directly from the Medicare Part D website—was shown in a grid on a computer screen and varied along six dimensions: plan name, estimated annual cost, monthly drug premium, annual deductible, coverage in the gap, and number of network pharmacies (see Figure 1A). The task required participants to move the computer mouse to “acquire” information concealed underneath the labelled boxes (see Figure 1B). Once they moved the cursor from one box to the next, the previous box closed and the new one opened. Participants completed two decision trials, choosing from either three or nine drug plans, presented in random order. We analysed data from the 70 participants who completed both trials (89.7% of the original sample), representing 122 decision trials.

#### Outcome measures

Participants’ decision quality was assessed by measuring whether they chose the lowest cost plan and determining the dollar amount of the loss if an alternative to the lowest cost plan was chosen. The lowest cost plan was defined as the plan with the lowest estimated annual cost (premiums + out-of-pocket) which can be found in the second row of Figure 1A and B. Although drug plan choice can be impacted by several preferences, for a number of reasons we chose to focus on estimated annual cost as the outcome. First, saving money is the most important factor in older adults’ decision about which drug plan to purchase [15]. Second, using estimated annual costs allowed us to compare apples to apples. Third, by focussing on a single attribute, we simplified the decision process and reduced the cognitive load needed to make the decision. If clinicians experienced difficulties making a decision based on a single attribute, it follows that making a decision incorporating multiple attributes would be more challenging.

#### Experimental condition

An indicator for whether the trial had three or nine drug plans to choose from was included in the analyses as a dichotomous variable.

#### Decision process and strategy covariates

Average time per acquisition (i.e., box opened), proportion of information reacquired, and search pattern were computed. Average time was defined as the total time in seconds divided by the total number of acquisitions. The proportion of information reacquired was defined as the proportion of boxes examined more than once divided by the total number of different boxes examined. Reacquisition ranged from 0 to 1. Search pattern, previously used in process-tracing studies [7,20], was defined as the relative degree to which individuals made alternative-based versus attribute-based decisions. More formally, search pattern was defined as the number of attribute-based transitions (e.g., moving the cursor from premiums to number of pharmacies within Plan A) subtracted from the number of alternative-based transitions (e.g., moving the cursor from Plan A to Plan B within the premium attribute) divided by the sum of attribute- and alternative-based decisions. Search pattern ranged from -1 to 1 with negative values indicating a more attribute-based strategy and positive values a more alternative-based strategy, and 0 indicating an even mix of both. Prior research with non-clinicians has shown that decision makers will attempt to reduce the cognitive demands of a choice task by comparing many attributes within an option, rather than an attribute across several alternative options - an inefficient approach to choosing when the choice objective relies on comparing a single attribute across alternatives [7,20].

#### Other covariates

We also controlled for participants’ amount of medical training (medical student or resident) and their numeracy skills. Numeracy was assessed using the Lipkus measure which has been validated to assess facility with probabilities and numbers among highly educated samples [24,25]. Previous work on highly educated samples has found the average numeracy score was 8.4 [25] with 32% of respondents answering all items corrects [24]. The measure consists mostly of questions on the calculation of basic probabilities and the ability to compare risks with fill in the blank responses allowing correct answers to be scored 1 and 0 otherwise. Participant performance on the numeracy scale was assessed by summarizing correct responses. The Lipkus measure has been used recently to assess the numeric ability of seniors [26], physician trainees [19], and practicing physicians [27].

### Statistical Analysis

Chi-square and *t* tests were used to evaluate unadjusted differences in decision quality, decision process, and strategy in the two conditions (three vs. nine plans). Multi-level regression was used to examine associations of the number of drug plan choices with the decision outcomes while controlling for observed confounders (i.e., decision process and strategy, amount of medical training, numeracy) and accounting for the within-subject repeated-measures design. Level 1 modelled information from each trial for each participant as separate observations. Level 2 included a random intercept term for each individual to capture differences among trials across participants.

To estimate the adjusted association of choice-set size with the odds of choosing the lowest cost plan, multi-level logistic models were used. Multi-level linear regression models were employed to estimate the adjusted associations of the experimental condition and the excess expenditure by the beneficiary if participants chose a plan other than the one that cost the least. Many participants chose the lowest cost plan, resulting in a number of zero values for the loss variable. As a sensitivity test, a panel data version of a generalized estimating equation (GEE) assuming a negative binomial distribution for the loss variable was estimated. The results of the two models were similar. For ease of interpretation, the multi-level linear model was chosen. An alpha level of .05 was used to determine the statistical significance of the model estimates.

## Results

Overall, fewer than half (48.3%) of the participants correctly identified the lowest cost drug plan, and the average excess premium payment incurred when choosing a higher cost plan was $97.62 per year (SD 100.51; Table 1). In regard to decision process and strategy measures, the average time spent acquiring a piece of information (i.e., the value of an attribute for a plan) was 0.84 s (SD 0.27). Very little information was reacquired once viewed (proportion reacquired = 0.09; SD 0.10), and participants employed an even mix of attribute- and alternative-based decision strategies (search pattern = 0.01; SD 0.65). Participants were highly numerate, scoring an average of 10.4 (SD 1.17) out of 11 possible points.

**Table 1:** Participant Characteristics (N=70 participants, 122 decision trials).

	Frequency or Mean (SD)	Three Plan Condition	Nine Plan Condition	P-value
Chose lowest cost plan	48.3%	67.3%	32.8%	<0.01
Dollar amount lost if lowest cost plan not chosen	97.62 (100.51)	60.00	128.51	<0.01
Average time per acquisition (seconds)	0.84 (0.27)	0.91	0.79	0.019
Proportion of information reacquired	0.09 (0.10)	0.08	0.09	0.886
Search pattern	0.01 (0.65)	-0.19	0.16	<0.01
Numeracy	10.4 (1.17)	10.36	10.43	0.746
Medical resident	13.9%	12.7%	14.9%	0.727

Before adjusting for decision process and strategy, numeracy and amount of medical training, participants were significantly more likely to identify the lowest cost plan when facing three versus nine choices (67.3% vs. 32.8%, p<0.01) and paid significantly less in excess premiums ($60.00 vs. $128.51, p<0.01). Compared to the three-plan condition, in the nine-plan condition participants spent significantly less time on average acquiring information on each attribute (p<0.05) and were more likely to employ a decision strategy that focussed on comparing alternate plans across a single attribute (search pattern, p<0.01).

After adjusting for decision process and strategy, numeracy, and medical training, the odds were 10.75 times higher (95% CI 2.36, 48.96, p<0.01, Table 2) that participants would choose the lowest cost Medicare Part D drug plan when facing three versus nine drug plans. There was a weak effect of more numerate participants having increased adjusted odds of choosing the lowest cost drug plan (adjusted odds ratio = 1.75; 95% CI 0.94, 3.29; p<0.10). The adjusted odds of choosing the lowest cost plan did not differ significantly by the average amount of time spent acquiring each piece of information, the proportion of information reacquired, the extent to which participants employed attribute- versus alternative-based decision strategies, or between medical students and residents.

**Table 2:** Adjusted Odds of Choosing the Lowest Cost Plan and Amount Lost if a Higher Cost Plan was Chosen (N=70 participants, 122 decision trials).

	Odds of Choosing Lowest Cost Plan	Dollar Amount Lost
	(95% CI)	(95% CI)
3 vs. 9 drug plans to choose from	10.75**	-68.51**
	(2.36, 48.96)	(-98.10, -39.22)
Average time per acquisition (seconds)	0.39	33.14
	(0.03, 5.08)	(-37.12, 103.40)
Proportion of information reacquired	0.10	76.40
	(0.01, 52.19)	(-99.89, 252.69)
Search pattern	0.89	7.13
	(0.35, 2.26)	(-17.64, 31.91)
Numeracy	1.75*	-7.34
	(0.94, 3.29)	(-23.85, 9.17)
Medical resident	1.16	-8.89
	(0.19, 7.10)	(-62.87, 45.09)

** p<0.05, *p<0.10

Finally, participants choosing from among three drug plans would pay $68.51 (95% CI -98.10, -39.22; p<0.01) less in excess premium payments compared to choosing from among nine plans, after adjustment. No significant associations were found between decision process and strategy, numeracy, or medical training covariates and the amount of excess premium paid.

## Discussion

We found that highly numerate medical students and residents chose the lowest cost plan 48% of the time. Moreover, the odds were about 10 times higher that they would choose the lowest cost prescription drug plans when faced with three plan options instead of nine. Our results here echo recent evidence from experiments and Part D enrollment data in which individuals were substantially better able to identify the cheapest insurance plan available when there were fewer choices [4,6–8]. This result is contrary to neoclassical economic theories of consumer behavior, in which more choice is always better, but consistent with Herbert Simon’s theory of bounded rationality, which recognizes the limits of human ability to comprehend vast amount of information [28]. Although policy makers and Medicare beneficiaries have expected physicians to play a key role in ensuring that beneficiaries make the right choice, both with regard to drug coverage and expenditures, our data question this assumption. Furthermore, if physicians, or in our sample, physician trainees, are facing difficulties with a simplified version of the Medicare choice sets, without having to integrate multiple factors, it seems unrealistic to assume that less educated and less numerate older adults will fare any better. Indeed, a previous study found a community-based sample of adults chose the lowest cost plan only 46% of the time and had 4 times the odds of choosing the lowest cost plan when given three rather than nine options [7]. Our results from physician trainees, moreover, are aligned with those of earlier studies showing that the majority of practicing clinicians found Medicare Part D too complicated, lacked familiarity with Part D formularies, and were hindered by its complex design when prescribing medications that were covered by their patients’ plan [18].

Price is one of the key concerns for Medicare beneficiaries and the chief factor in their decision about which drug plan to purchase [15]. Our study shows that physician trainees had greater difficulties identifying the cheapest drug plan when faced with a large number of options. Indeed, our data indicate that facing nine plans had a debilitating effect on their ability to pick the cheapest drug plan, with an average loss of about $70 per plan. Our results, however, are probably conservative, as the average number of Medicare Part D plans is over 30 [2]. As our investigation focussed on decisions based on a single choice attribute, we can assume that clinicians will face an even greater challenge in selecting the best plan when more choice dimensions must be considered (e.g., price, pharmacy preference, overall plan quality).

Interestingly, in the present study medical students and residents utilized a more efficient search strategy when facing nine compared to three plans in that they were more likely to focus their decision on comparing an attribute across alternatives. Yet, despite using a more efficient search strategy, the decision outcome was worse in the choice-rich environment (nine plans), a further indication of the deleterious effect of too much choice. The finding that physician trainees used better search strategies, however, was somewhat counter to results of previous studies with non-clinicians. A number of studies [19–21], for instance, have shown that as task complexity and time pressure increase, individuals tend to use non-compensatory decision strategies. Indeed, an earlier study using a community sample of adults [7] reported that as choice size increased from three to nine plans, participants tended to employ a more attribute-based search strategy. However, the present decision-process results for physician trainees do match earlier findings for non-clinicians [7,8] where an increase in task complexity was associated with reduced time spent on each acquisition. The present work provides a richer and more complex picture of physician trainees’ decision-making processes and strategy selection within the Medicare Part D environment.

This study is not without limitations. First, the sample was one of convenience and our sample of medical students and residents may not generalize to all physician trainees. In regard to whether our findings generalize to all physician trainees at the study site, the demographic data collected from participants included age, whether they were a medical student or resident, what year they were in, and what field they expected to work during the five years after completing their clinical training. The majority of participants (86%) were medical students rather than residents. The distribution of first, second, third, and fourth year medical students in our sample was consistent with medical school admissions data at the study site suggesting respondents to our study were not systematically different in training than non-respondents.

Second, physician trainees’ substantive knowledge of and experience with Medicare Part D are certain to be limited. It is possible that more practicing physicians dealing with patients would perform better. Nonetheless, we argue physician trainees should possess the cognitive abilities to evaluate the drug plan information, compare attributes across plans, and make an appropriate choice. If they do not, as our data suggest, medical education policymakers should consider incorporating these skills into the medical curricula as some have argued [29,30]. Third, the decision was hypothetical, with no impact on patients or the physician trainees who participated. Finally, currently there is lack of data on what information seniors are using when making their purchasing decision as well as the extent to which clinicians are actually helping their older patients make decisions about Medicare drug plans. Future work in this area is critical to direct policy to bolster the avenues of communication seniors are currently using or steer seniors towards better information sources.

The study has a number of policy implications. Physicians may not be best equipped to assist their patients in choosing one Medicare Part D plan over another. Both the Medicare plan finder and Medicare help line provide seniors with personalized information on current costs of all plans available in their market given their expected prescription drug needs. Physicians’ role in senior patients’ Part D choices may be most productive when assisting seniors with forecasting their expected medication needs in the coming plan year and then referring them to the Medicare website or helpline as recent research has found pharmacists are doing [5]. In regard to the Part D decision environment, recent evidence questions the value of strictly limiting choice in Medicare Part D plans [31]. However, policy makers might consider revising the Medicare Part D website by presenting beneficiaries with less information about each plan. As has been nicely argued [6], “beneficiaries need more targeted assistance from the government to help them choose plans, such as customized communications about the most cost-effective plans that would cover their medication needs.” Programs by Medicare to selectively contract with a subset of the highest value plans have been proposed recently [32], as have interventions to allow for easier comparison between the drug plans by increasing the saliency of plan differences by using standardizing drug plans and decreasing reliance on numeric comparisons across plans by employing symbolic representation of plan information [8]. Others have argued that an even more effective approach would be for Medicare to estimate which drug plan would be cheapest for beneficiaries given their current drug regimens and automatically enroll them [33] - a good example of a policy “nudge” [34]. Furthermore, our results should inform the development and design of health insurance exchanges programs [35]; given the possibility that they will also be rich in choice, there are concerns that consumers choosing in these environments will face similar struggles.
